# Delta-subunit-containing GABA_A_-receptors mediate tonic inhibition in paracapsular cells of the mouse amygdala

**DOI:** 10.3389/fncir.2014.00027

**Published:** 2014-03-25

**Authors:** Anne Marowsky, Kaspar E. Vogt

**Affiliations:** ^1^Institute of Pharmacology and Toxicology, University of ZurichZurich, Switzerland; ^2^International Institute for Integrative Sleep Medicine, University of TsukubaTsukuba, Japan

**Keywords:** paracapsular cells, THIP, tonic inhibition, delta-GABAA receptor, Amygdala

## Abstract

The intercalated paracapsular cells (pcs) are small GABAergic interneurons that form densely populated clusters surrounding the basolateral (BLA) complex of the amygdala. Their main task in the amygdala circuitry appears to be the control of information flow, as they act as an inhibitory interface between input and output nuclei. Modulation of their activity is thus thought to affect amygdala output and the generation of fear and anxiety. Recent evidence indicates that pcs express benzodiazepine (BZ)-sensitive GABA_A_ receptor (GABA_A_R) variants containing the α2- and α3-subunit for transmission of post-synaptic currents, yet little is known about the expression of extrasynaptic GABA_A_Rs, mediating tonic inhibition and regulating neuronal excitability. Here, we show that pcs from the lateral and medial intercalated cell cluster (l- and mITC, respectively) express a tonic GABAergic conductance that could be significantly increased in a concentration-dependent manner by the δ-preferring GABA_A_R agonist THIP (0.5–10 μM), but not by the BZ diazepam (1 μM). The neurosteroid THDOC (300 nM) also increased tonic currents in pcs significantly, but only in the presence of additional GABA (5 μM). Immunohistochemical stainings revealed that both the δ-GABA_A_R and the α4-GABA_A_R subunit are expressed throughout all ITCs, while no staining for the α5-GABA_A_R subunit could be detected. Moreover, 1 μM THIP dampened excitability in pcs most likely by increasing shunting inhibition. In line with this, THIP significantly decreased lITC-generated inhibition in target cells residing in the BLA nucleus by 30%. Taken together these results demonstrate for the first time that pcs express a tonic inhibitory conductance mediated most likely by α4/δ-containing GABA_A_Rs. This data also suggest that δ-GABA_A_R targeting compounds might possibly interfere with pcs-related neuronal processes such as fear extinction.

## Introduction

The intercalated paracapsular cells form clusters of dopamine-D1 and μ-opioid receptor-rich GABAergic interneurons, which comprise several subgroups surrounding the basolateral (BLA) amygdala (Millhouse, 1986; Nitecka and Ben-Ari, 1987; Busti et al., 2011). Of these subgroups, the medial intercalated cell cluster (mITC) is located between the lateral (LA)/(BLA) and the central nucleus (CeA) of the amygdala, while the lateral counterpart (lITC) is located along the external capsule. As paracapsular intercalated cells (pcs) are innervated by projections from the medial prefrontal cortex, they are thought to provide cortical control over the amygdala (McDonald et al., 1996; Quirk et al., 2003; Berretta et al., 2005). In particular, pcs from the lITC provide feedforward-inhibition onto BLA cells, while those of the mITC target cells in the CeA (Royer et al., 1999; Marowsky et al., 2005). Furthermore, a thorough interaction between neurons of all ITCs exists and notably mITC neurons were reported to project to other areas outside the amygdala (Busti et al., 2011; Palomares-Castillo et al., 2012).

The amygdala plays a central role in fear and anxiety (Ledoux, 2000), and information flow through its various nuclei is well-established, with the LA nucleus generally considered the main input and the CeA the main output nucleus (Ehrlich et al., 2009). In the amygdala circuitry, the ITC neurons fulfill the role of an inhibitory gate under cortical control (Pare et al., 2003). When sufficiently activated by converging excitatory input from BLA nucleus and cortex, mITC neurons seem capable of depressing fear reaction-eliciting signaling from the CeA (Royer et al., 1999). Furthermore, mITC neurons play a central role in fear extinction (Amano et al., 2010). Indeed, the selective targeting and destruction of the mITC cluster by a toxin led to a decrease in freezing displayed by lessoned animals, which correlated with the number of surviving cells (Likhtik et al., 2008). At a cellular level, the combined excitatory inputs from BLA neurons and infralimbic cortex on mITC neurons are thought to produce sufficient depolarization to enable the induction of extinction-related plasticity (Royer and Pare, 2002).

Excitability of neurons is crucially determined by GABAergic inhibition. Two types of inhibition are mediated by GABA_A_ receptors (GABA_A_Rs), named phasic and tonic inhibition (Farrant and Nusser, 2005). The former is characterized by synaptically released GABA that transiently activates post-synaptic GABA_A_Rs, producing brief inhibitory post-synaptic currents (IPSCs). By contrast, tonic inhibition is the result of ambient GABA in the extracellular space that persistently activates mostly extrasynaptically located GABA_A_Rs (Belelli et al., 2009). All GABA_A_Rs are pentameric Cl^−^-permeable ion channels with subunits drawn from seven subfamilies (α1–6, β 1–3, γ1–3, δ, π, θ, ε) (Whiting et al., 1999). While synaptically activated GABA_A_Rs are typically formed from 2 α-subunits, 2 β-subunits and 1 γ-subunit, extrasynaptically located GABA_A_Rs carrying tonic currents most often contain the α4/δ subunits (e.g., in thalamus and in dentate gyrus granule cells) or the α6/δ subunits (exclusively expressed in the cerebellum) (Rossi and Hamann, 1998; Hamann et al., 2002a,b; Borghese et al., 2006; Chandra et al., 2006). Another tonically activated GABA_A_Rs subtype contains the α5 subunit expressed by CA1 hippocampal pyramidal cells (Caraiscos et al., 2004).

In the amygdala α3-containing GABA_A_Rs are probably the dominant receptor type generating tonic currents in BLA principal cells (Marowsky et al., 2012). Immunohistochemical stainings and functional evidence suggest that pcs express α2- and α3-containing GABA_A_Rs (Marowsky et al., 2004; Geracitano et al., 2012). Recently, it has been shown that pcs also express a tonic GABAergic current, most probably not mediated by α3-GABA_A_Rs as the α3 GABA_A_Rs-selective agonist TP003 failed to modulate it (Marowsky et al., 2012). In general, GABA_A_Rs carrying the α1, α2, α3 or α5-subunit in combination with the γ2-subunit are benzodiazepine (BZ)-sensitive, while δ-containing GABA_A_Rs are distinctly insensitive to the positive allosteric modulation by these compounds (Hamann et al., 2002a; Farrant and Nusser, 2005). Pharmacologically, δ-GABA_A_R mediated currents can be modulated by neurosteroids such as THDOC or THP (Brown et al., 2002; Stell et al., 2003; Mtchedlishvili and Kapur, 2006) and by the δ-preferring agonist THIP (Brown et al., 2002; Storustovu and Ebert, 2006; Bonin et al., 2011). Applying immunohistochemical and electrophysiological techniques, here we aim to investigate which GABA_A_R subtype(s) underlie(s) tonic currents in pcs of the mouse amygdala.

## Materials and methods

### Animals

Acute slices for electrophysiology were prepared from 3 to 5 week-old male GAD67-GFP mice obtained from Yuchio Yanagawa (Gunma University, Maebashi City, Japan) (Tamamaki et al., 2003). These mice are heterozygous for the altered GAD67-GFP allele and were maintained on a C57BL/6J background. C57BL/6J mice for breeding and immunohistochemistry were obtained from Harlan Laboratories.

### Electrophysiology

GAD67-GFP mice were deeply anaesthetized with isoflurane, decapitated and the brain was quickly removed. After mounting on a HM 650V Microm (Thermoscientific), the brain was cut into 300 μm thick coronal slices. Ice-cold artificial cerebro-spinal fluid (ACSF) was used as cutting solution, containing (in mM) 125 NaCl, 26 NaHCO_3_, 1.25 NaH_2_PO_4_(H_2_O), 2.5 KCl, 1 MgCl_2_,11 glucose and 2.5 CaCl_2_, pH 7.4 in 95% O_2_ and 5%CO_2._ Slices were maintained on a nylon mesh in a closed beaker filled with ACSF warmed to 32°C, and were allowed to equilibrate for at least 30 min before the start of experiment. In the recording chamber, slices were submerged and superfused (1–2 ml/min at 32 ± 2°C) with ACSF. For visualization of the GFP-expressing neurons in slices an upright microscope (Olympus BX51Wl) equipped with Nomarski differential interference contrast optics, an infrared videoimaging camera (VX55 Till Photonics), and a standard 100 W tungsten lamp connected to an epifluorescence system were used. The GABA_B_ receptor blocker CGP 54626 (0.5 μ M) and kynurenic acid (2.5 mM), which blocks excitatory synaptic transmission, were routinely added to ACSF. For recordings of tonic currents, mIPSCs (in the presence of 1 μM TTX added to ACSF) as well as stimulated IPSCs, patch pipettes with tip resistances of 4–8 MΩ were filled with an internal solution containing the following (in mM): 100 CsCl, 40 HEPES, 2 MgATP, 0.3 NaGTP, and 0.1 EGTA. The pH was adjusted to 7.3 with CsOH, the osmolarity to 290–300 mOsm. Cells were voltage-clamped at −70 mV, so GABA_A_R-mediated currents were inward. For experiments studying the bicuculline and THIP effect on excitability of pcs, pcs were held in current clamp and patch pipettes were filled with an internal solution containing (in mM) 130 K gluconate, 1 EGTA, 10 HEPES, 5 MgATP, 0.5 NaGTP, and 5 NaCl. The pH was adjusted to 7.3 with KOH and osmolarity to 290–300 mOsm. Pcs were injected with current pulses ranging from −10 pA to + 100 pA as indicated. Evoked responses at 0.1 Hz were elicited by a patch electrode filled with ACSF. The tip was positioned either at the dorsal end of a lateral paracapsular cell cluster adjacent to the external capsule or in the BLA nucleus. Diazepam was provided by Hoffmann-La Roche. THIP [4,5,6,7-Tetrahydroisoxazolo(5,4-c)pyridin-3-ol] and bicuculline were purchased from ANAWA (S)witzerland). THDOC (Tetrahydrodeoxycorticosterone or 3α,21-dihydroxy-5α-pregnan-20-one) was purchased from Steraloids (Newport, USA). All other chemicals were purchased from Sigma/Fluka or Tocris Bioscience.

### Immunohistochemistry

Distribution of the GABA_A_R subunits α4, α5, and δ and GAD-GFP positive neurons was visualized by immunoperoxidase and -fluorescence staining of coronal sections from perfusion-fixed tissue of 4- to 12-week-old C57 Bl/6J and GAD67-GFP mice. The following antibodies were used: homemade guinea pig anti-α5 (Fritschy and Mohler, 1995), rabbit anti-α4 (PhosphoSolutions), rabbit anti-δ (Millipore Bioscience Research Reagents), and mouse anti-GFP (Novus Biologicals). Specificity of antibodies directed against GABA_A_R subunits had been verified before in the respective KO mouse (data not shown). Mice were perfused with 4% paraformaldehyde in 0.15 m sodium phosphate buffer, pH 7.4. Brains were post-fixed for 3 h, incubated overnight and cryoprotected in 30% sucrose, then frozen and sectioned on a sliding microtome (40 μm). For immunofluorescence stainings sections were incubated overnight at 4°C with primary antibody in Tris buffer containing 2% normal goat serum and 2% Triton-100. After rinsing extensively they were incubated with secondary antibodies conjugated to Cy3 (1:500; Jackson ImmunoResearch Laboratories). Sections were mounted and coverslipped with fluorescence mounting medium (Dako, Denmark). For immunoperoxidase stainings, biotinylated secondary antibodies (1:300; Jackson ImmunoResearch, West Grove, PA, USA) were applied for 30 min, followed by Vectastain elite kit processing (Vector Laboratories, Burlingame, CA, USA) and incubated with diaminobenzidine as chromogen. Sections were mounted onto gelatin-coated glass slides, air-dried, dehydrated, and coverslipped with Eukitt. Images from immunoperoxidase staining were digitized with a high-resolution camera and processed using the software Mosaic (ExploraNova, LaRochelle, France). High-magnification images of immunofluorescence staining were acquired by laser scanning confocal microscopy (Zeiss LSM 510 META). Digital images were processed using the software Imaris (Bitplane, Zurich, Switzerland).

### Data analysis and statistics

Data were recorded with a Multiclamp 700 B amplifier (Molecular Devices), filtered at 3 kHz, and digitized at 20 kHz (A/D hardware, National Instruments). In all experiments, series resistance was monitored throughout the experiment by applying a hyperpolarizing pulse of 10 mV; if it changed by >20%, data were not included in the analysis. Data were acquired and analyzed with IGOR Pro software (Wave Metrics). Spontaneous events were detected with the Mini Analysis Program (Synaptosoft). For IPSC kinetics, averages of 30–100 sIPSCs per cell and condition were peak scaled and fit with both mono-

$$I(t)=A*\text{exp}(-t/\tau_{\text{total}})$$

and double-exponential equations

$$I(t)=A_{1}*\;(\text{exp}(-t/\tau_{1}))+A_{2}*\;[\text{exp}(-t/\tau_{2})]$$

with A the amplitude and τ_total_ the overall time constant for mono-exponential fit and *A*_1_ and *A*_2_ as the fast and slow component amplitudes and τ_1_ and τ_2_ as their respective time constants for double-exponential fits.

Tonic GABA_A_R-mediated current was defined as the shift in inward holding current (*I*_hold_) after application of the GABA_A_R blocker bicuculline and measured as described previously (Glykys et al., 2007; Krook-Magnuson et al., 2008; Marowsky et al., 2012). In short, 1 s streams of sIPSCs were sampled every 10 s. Traces were transcribed into all-point histograms, and points that fell on sIPSCs were discarded. The average values for baseline were calculated for six 1 s epochs before and for the bicuculline effect for six 1 s epochs after application of the blocker. A Gaussian distribution was fit to the all-points histogram, with the peak of the distribution determining the mean current for that sample. Total tonic currents were calculated from the difference of baseline and bicuculline mean holding current or from drug baseline and bicuculline mean holding current, respectively. Cell capacitance was calculated from the current transient obtained by giving a hyperpolarizing pulse of 10 mV at the beginning of each 1 s epoch. Results of several experiments are reported as average ± s.e.m. Simple and pairwise comparisons were performed with the appropriate two-tailed Student's *t*-test or a One-Way ANOVA followed by Bonferroni *post-hoc* test.

## Results

**Figure 1:**
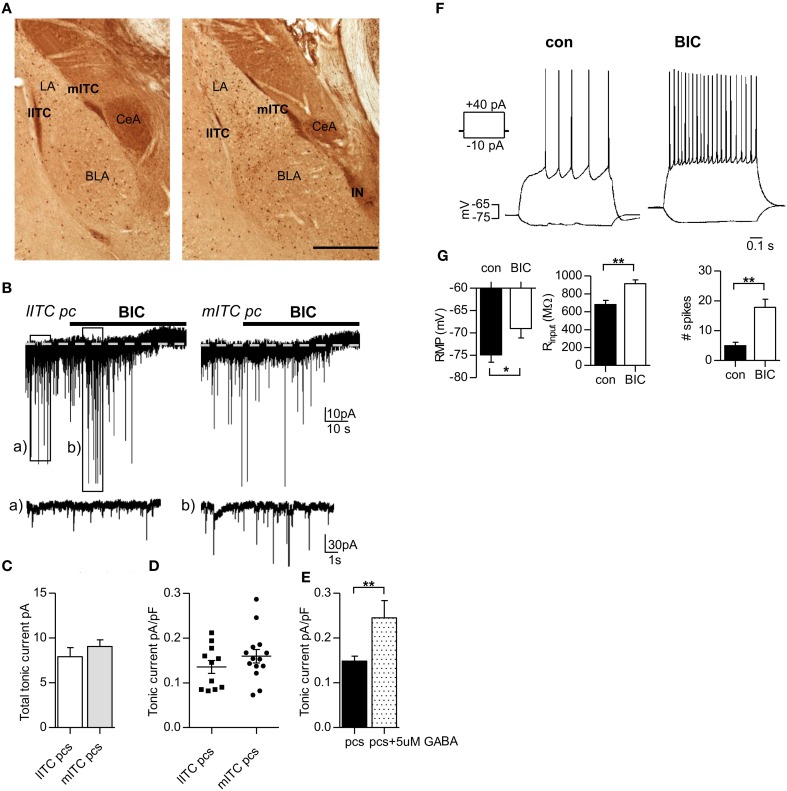
**Application of the GABA_A_R blocker bicuculline reveals a tonic conductance in lITC and mITC pcs that is sufficiently large to affect excitability in these cells. (A)** Immunostaining against GFP in a rostral (left) and a more caudal (right) fixated slice from GAD67-GFP mice. All GABAergic interneurons are labeled dark brown. The pcs form densely packed clusters along the lateral and medial border of the LA/BLA nucleus, which are termed lITC, mITC, and IN, respectively. **(B)** Representative traces from a lITC pc and a mITC pc, recorded under control conditions (*V*_*h*_ = −70 mV, 0.5 μM CGP54626, 2.5 mM kynurenic acid). GABAergic currents are inward. Black bars indicate wash-in of bicuculline (BIC, 25 μ M), leading to an outward shift in holding current. Note that under bicuculline sIPCS amplitude and frequency can transiently increase as shown in **(Bb)**, probably due to increased spiking from other pcs. **(C)** Summary graph of total tonic current in lITC and mITC pcs, calculated as the difference in I_hold_ before and after bicuculline addition. **(D)** Summary graph of tonic currents normalized to cell size in lITC and mITC pcs, showing no significant difference. **(E)** Summary graph of tonic currents in pcs with and without the addition of 5 μ M GABA. **(F)** Representative graphs of a pc held in current clamp and injected with a −10 and +40 pA pulse for 0.8 s before (control) and after bath-application of bicuculline (25 μ M). Recordings were carried out in the presence of 0.5 μM CGP54626 and 2.5 mM kynurenic acid. Under bicuculline the number of spikes increases markedly, accompanied by an upward shift in RMP (depolarization) and an increase in R_input,_ calculated from the −10 pA injection step. **(G)** Summary graphs for the effect of bicuculline on RMP, R_input_, and number of spikes in pcs. BLA, basolateral nucleus of the amygdala; lITC, lateral intercalated cell cluster; LA, lateral nucleus of the amygdala; mITC, medial intercalated cell cluster; IN, main intercalated nucleus; pcs, paracapsular cells. Scale bar in **(A)** 500 μm applies to both panels. **(C,E)** Unpaired Student's *t*-test; **(G)** Paired Student's *t*-test, ^*^p < 0.01 and f ^**^p < 0.01 Exact values for *p* and *n* are given in the text.

Application of the GABA_A_R blocker bicuculline (25 μ M) revealed a tonic inhibitory current in pcs of lITC and mITC, evidenced by an outward shift in holding current I_hold_ (lITC pcs 7.6 ± 0.9 pA, mITC pcs 9.1 ± 0.7 pA) (Figures 1A–C). Tonic currents were normalized to cell size (lITC pcs 55.3 ± 2.2 pF, *n* = 11, vs. mITC pcs 58.1 ± 3.5 pF, *n* = 14, *p* = 0.531 unpaired Student's *t*-test) and no difference in normalized tonic current was detected between neurons of the two clusters (lITC pcs 0.136 ± 0.016 pA/pF, *n* = 11, vs. mITC pcs 0.157 ± 0.015 pA/pF, *n* = 14, *p* = 0.302, unpaired Student's *t*-test) (Figure 1D). Thus, results for neurons from lITC and mITC were pooled from here on and cells are generally referred to as pcs unless otherwise stated. GABAergic tonic currents in pcs could be detected in the absence of any additional GABA or GABA uptake inhibitor. Yet tonic currents could still be strongly increased by adding additional GABA to the bath solution. Indeed, in the presence of 5 μM GABA, the bicuculline-sensitive current normalized to cells size increased significantly in pcs from 0.148 ± 0.011 pA/pF, *n* = 25, to 0.245 ± 0.017 pA/pF, *n* = 6, (*p* = 0.00164, unpaired Student's *t*-test) (Figure 1E).

Next, we wanted to know whether GABAergig tonic currents in pcs are sufficiently large to affect excitability in these cells. As pcs are only rarely active in brain slices, action potentials were evoked by stimulating pcs with depolarizing current steps, which were repeated with the same magnitude after washing in of bicuculline (25 μ M). In addition, a −10 pA-step was given in the absence and presence of bicuculline to calculate the input resistance of the cell (R_input_). Washing in of bicuculline significantly increased the number of action potentials fired, accompanied by a marked increase in R_input_ and resting membrane potential (RMP) of the cell. [Number of action potentials (+40 pA injection) control: 5.0 ± 1.1 vs. bicuculline: 17.8 ± 2.8, *p* = 0.0083, R_input_ control: 682 ± 45 MΩ vs. bicuculline: 914 ± 43 MΩ, *p* = 0.0091; RMP control: −74.9 ± 1.6 mV vs. −69.0 ± 2.1 mV, *p* = 0.0148, for all comparisons paired Student's *t*-test were applied, *n* = 6] (Figures 1F,G). These findings indicated that GABAergic tonic currents are indeed effective in modulating the excitability of pcs. However, these results should be interpreted with caution, as bicuculline blocks phasic and tonic inhibition in a non-selective manner.

**Figure 2:**
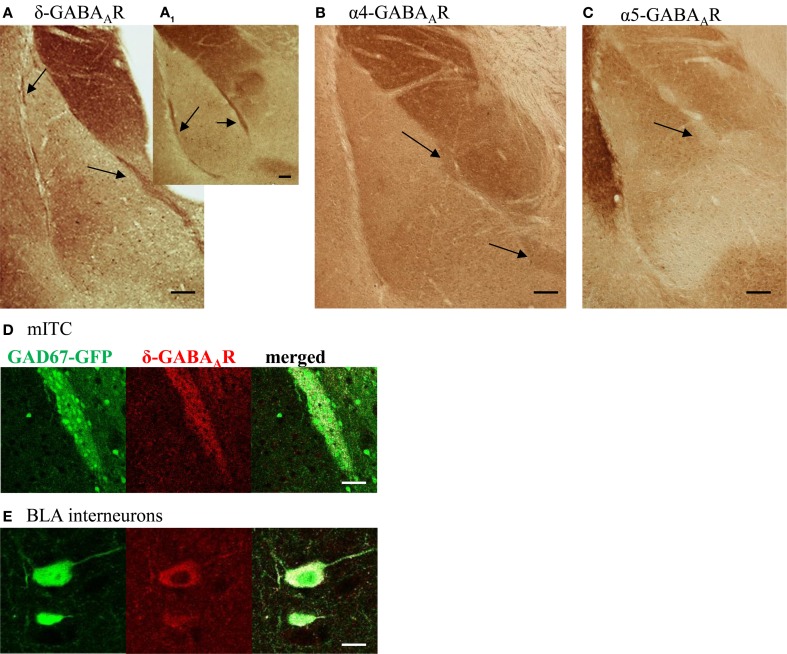
**Pcs are stained positive for the δ- and α4-GABA_A_R subunit, but not for the α5- GABA_A_R subunit. (A–C)** Coronal amygdala sections from WT mice stained for the GABA_A_R subunits δ, α4, and α5. Arrows point to intercalated cell clusters. Note the intense δ-IR throughout intercalated cells clusters on a caudal **(A)** and rostral **(A**_1_**)** slice. In addition to pcs, single neurons in LA and BLA nucleus are stained δ-positive, visible as brown dots. Pcs also show α4-GABA_A_R IR as seen in **(B)**, but are negative for α5-GABA_A_R IR. Note the strong α5-GABA_A_R IR in the adjacent endopiriform nucleus. **(C)**. All scale bars **(A–C)** 0.1 mm **(D)** δ-GABA_A_R labeling (red, middle panel) surrounds and partly co-localizes with GFP-labeled (green, left panel) pcs forming the mITC in slices from GAD67-GFP mouse. Co-localization is shown in white in the right panel. Scale bar 50 μm **(E)** δ-GABA_A_R labeling (red, middle panel) colocalizes with GFP-labeling (green, left panel) in a subset of large GABAergic interneurons, frequently found in the BLA nucleus. Co-localization is shown in white in the right panel. Scale bar 10 μm.

Typically, tonic currents are carried by α4/δ or α5-containing GABA_A_Rs. Immunohistochemical stainings using respective α4-, α5,- and δ-antibodies on coronal mouse brain slices revealed α4- and δ-GABA_A_R immunoreactivity (IR) throughout all ITCs (Figures 2A,B). In contrast, α5-GABA_A_R IR could not be detected in any of the clusters (Figure 2C). Interestingly, δ-GABA_A_Rs-positive cells are rare in LA and BLA nucleus: in addition to pcs only a subset of large GABAergic interneurons, identified by co-localization of GFP and δ-GABA_A_R IR in slices from GAD67-GFP mice, appears to express δ-containing GABA_A_Rs (Figures 2D,E).

**Figure 3:**
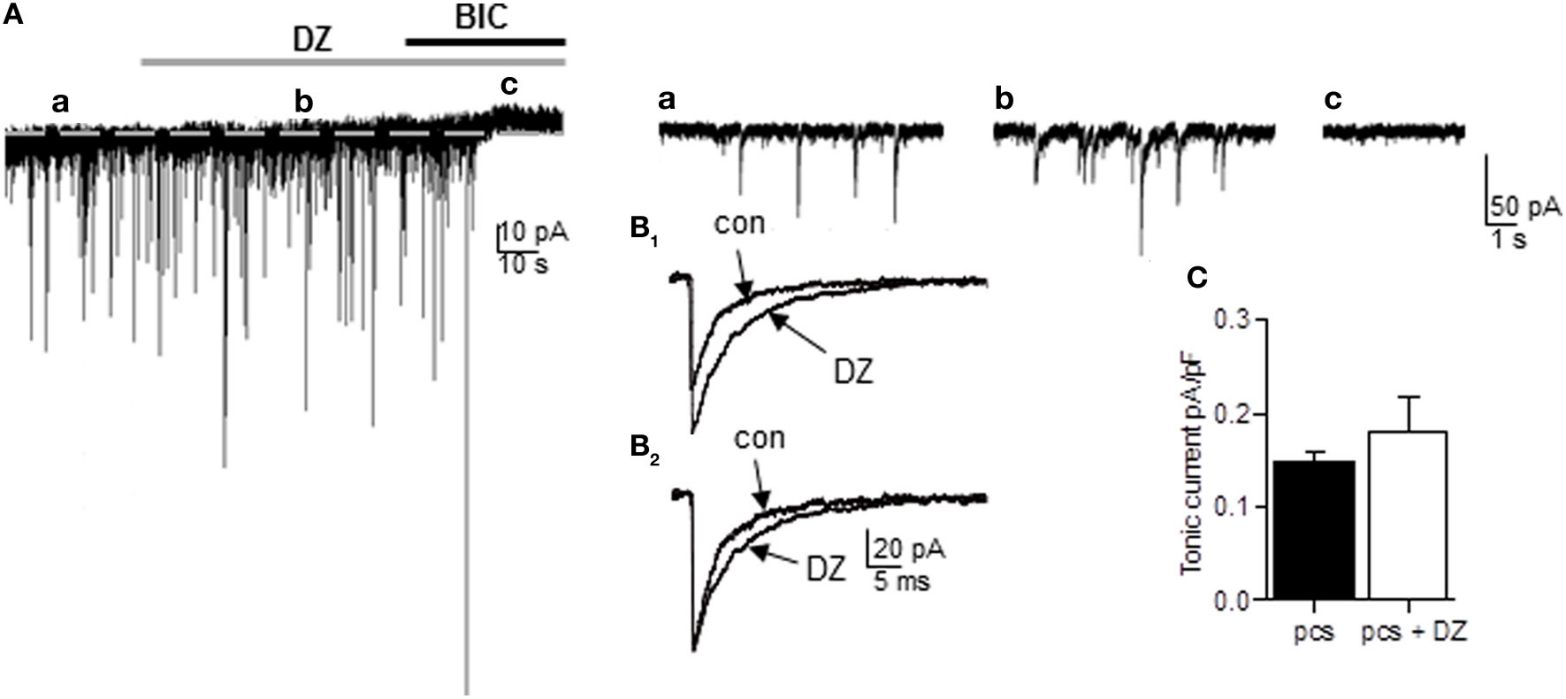
**The non-selective BZ diazepam fails to increase tonic current in pcs. (A)** Representative trace of a pc before and after bath application of diazepam (DZ, 1 μ M, gray bar) followed by bath application of bicuculline (BIC, 25 μ M, black bar). For the amplitude of tonic current, the difference in I_hold_ under diazepam **(b)** and bicuculline **(c)** was calculated. Note the absence of any shift in I_hold_ under diazepam in **(b)**. Right panel: Enlarged traces before **(a)** and after application of diazepam **(b)** and bicuculline **(c)**. There is no apparent increase in baseline noise visible under diazepam, but an increase in sIPSC amplitude **(B**_1_**)** Enlarged traces of average sIPSCs recorded before and after diazepam application. Note the diazepam-induced increase in amplitude. **(B**_2_**)** Same as in **(B**_1_**)**, but sIPSCs were peak-scaled so only the diazepam-typical increase in ISPC decay time is apparent. **(C)** Summary graph of diazepam effect on tonic currents in pcs. Exact values for *p* and *n* are given in the text.

In principle, all of the GABA_A_Rs variants expressed by pcs could mediate tonic currents in these cells. GABA_A_Rs carrying the α2- or α3-subunit in combination with the γ2-subunit should be benzodiazepine-sensitive such that respective currents are markedly potentiated by the application of non-selective BZ such as diazepam. We therefore recorded from pcs and bath-applied 1 μM diazepam and subsequently bicuculline (25 μM) (Figure 3A). Diazepam clearly led to potentiation of sIPSC amplitude and increase in sIPSC decay time, confirming the presence of synaptically located diazepam-sensitive GABA_A_Rs, which are most likely α2- and α3-containing GABA_A_Rs (Geracitano et al., 2012) (see Table 1, Figures 3A,B**_1_**,B**_2_**). However, in only 3 out of 10 cells a shift of holding current was observable after diazepam application with the result that on average no significant change in GABAergic tonic current could be observed (diazepam: 0.169 ± 0.025pA/pF, *n* = 10, vs. control 0.148 ± 0.011 pA/pF, *n* = 25; *p* = 0.142, unpaired Student's *t*-test) (Figure 3C). This finding suggested that α2 and/or α3-containing GABA_A_Rs contribute only to a limited degree to tonic currents in pcs, consistent with recent results obtained with the α3-selective agonist TP003 (Marowsky et al., 2012).

**Table 1:** **Drug effects on IPSC parameter**.

	**IPSC parameter**												
	**Frequency (Hz)**		**Amplitude (pA)**		**Rise time (ms)**		**τ1(ms)**		**τ2(ms)**		**τ total(ms)**		**Effect on tonic current**
	**Control**	**Drug**	**Control**	**Drug**	**Control**	**Drug**	**Control**	**Drug**	**Control**	**Drug**	**Control**	**Drug**	
**PARACAPSULAR CELLS PCS**													
1 μ M DZ (10)	1.3 ± 0.2	1.6 ± 0.2	37.4 ± 7.7	45.3 ± 9.0^*^	1.3 ± 0.3	1.6 ± 0.3	12.6 ± 1.7	18.1 ± 1.7^*^	41.9 ± 1.2	76.5 ± 8.9^*^	21.8 ± 1.8	32.0 ± 2.5^***^	no
0.5 μ M THIP (5)	2.3 ± 0.7	2.1 ± 0.6	33.2 ± 6.8	31.6 ± 6.0	0.9 ± 0.2	0.8 ± 0.1	9.4 ± 2.4	11.7 ± 3.8	39.1 ± 8.1	36.7 ± 10.3	21.2 ± 2.8	20.5 ± 6.1	no
1 μ M THIP (12)	2.1 ± 0.4	1.9 ± 0.3	36.9 ± 5.3	31.3 ± 4.6	1.0 ± 0.1	0.9 ± 0.2	9.9 ± 2.2	9.4 ± 2.2	36.7 ± 5.7	31.9 ± 5.8^*^	20.2 ± 2.3	17.9 ± 3.3	yes
300 nM THDOC (9)	1.6 ± 0.3	1.2 ± 0.1	36.4 ± 3.0	33.6 ± 2.7	1.3 ± 0.3	0.9 ± 0.1	10.4 ± 1.2	14.8 ± 2.4	39.1 ± 6.5	55.3 ± 8.2^*^	22.6 ± 2.5	33.5 ± 3.8^**^	no
300 nM THDOC + 5 μ M GABA (6)	1.7 ± 0.4	1.5 ± 0.3	35.1 ± 2.6	34.9 ± 3.8	1.2 ± 0.4	1.0 ± 0.3	10.1 ± 1.9	13.5 ± 2.9	38.1 ± 7.4	59.1 ± 9.1^*^	21.1 ± 3.3	31.5 ± 3.1^**^	yes
**PRINCIPAL CELLS BLA**													
1 μ M THIP+TTX (5)	2.1 ± 0.1	2.2 ± 0.2	28.1 ± 0.4	27.7 ± 0.8	0.7 ± 0.1	0.6 ± 0.1	8.6 ± 0.4	7.7 ± 0.6	31.1 ± 7.1	26.9 ± 1.0	13.6 ± 0.2	14.2 ± 0.5	no
1 μ M THIP (12)	2.7 ± 0.6	2.6 ± 0.6	30.2 ± 2.6	33.0 ± 3.7	0.9 ± 0.1	0.9 ± 0.1	7.6 ± 0.8	7.8 ± 0.9	17.5 ± 2.0	18.2 ± 2.8	11.5 ± 1.0	10.9 ± 1.0	no

Drug values (mean ± s.e.m.) significantly different from control values are indicated with

* p < 0.05,

** p < 0.01 and

*** p < 0.001 (Student's paired t-test).

Numbers of recorded neurons are given in parenthesis.

**Figure 4:**
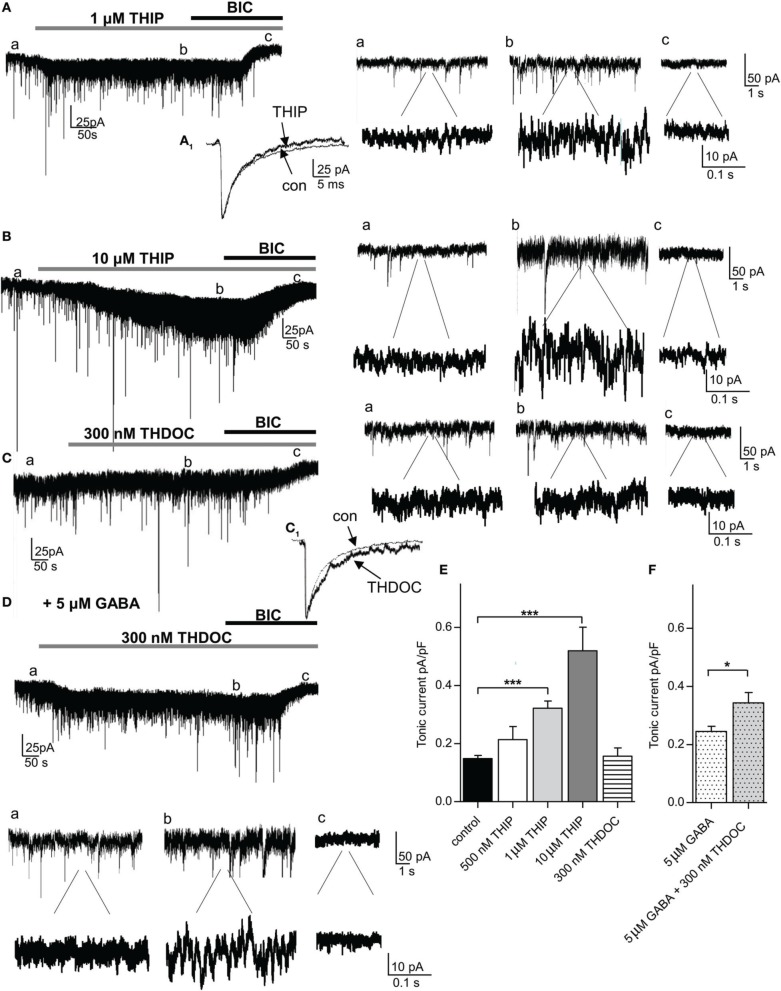
**The δ-preferring agonist THIP increases tonic currents in a concentration-dependent manner in the absence of any additional GABA, while the neurosteroid THDOC requires the presence of 5 μ M GABA to potentiate tonic currents in pcs**. **(A,B)** left panel: Representative traces of a pc before and after bath application of THIP (1 and 10 μ M, gray bar) followed by bath application of bicuculline (BIC, 25 μ M, black bar). Note the shift in I_hold_ under THIP in **(Ab,Bb)**. For the amplitude of tonic current, the difference in I_hold_ under THIP **(b)** and bicuculline **(c)** was calculated. **(A**_1_**)** Enlarged traces of average sIPSCs recorded before and after application of 1 μ M THIP. THIP accelerates IPSC kinetics, as it shortens the tail current. Right panel: Enlarged traces before **(a)** and after application of 1 and 10 μ M THIP **(b)** and bicuculline **(c)** in two magnifications. Note the clear increase in baseline noise in presence of 1 and 10 μ M THIP. **(C)** Representative trace of a pc before and after bath application of THDOC (300 nM, gray bar) followed by bath application of bicuculline (25 μ M, black bar). There is no shift in I_hold_ visible under THDOC in (**Cb)**. (**C**_1_**)** Enlarged traces of average sIPSCs recorded before and after application of 300 nM THDOC. Note that the sIPSC decay time is prolonged in presence of the neurosteroid. Right panel: Enlarged traces before **(a)** and after application of THDOC **(b)** and bicuculline **(c)** in two magnifications. **(D)** Same as in **(C)** but prior to bath application of THDOC (300 nM) 5 μ M GABA were washed in. Under these conditions there is a clear shift in I_hold_ visible under THDOC as seen in (**Db)**. Below: Enlarged traces before **(a)** and after application of THDOC **(b)** and bicuculline **(c)** in two magnifications. Note the increase in baseline noise in presence of GABA and THDOC **(Da,Db)**. **(E)** Comparison of THIP and THDOC effects on tonic currents in pcs in the absence of any additional GABA. **(F)** Comparison of THDOC (300 nM) effect on tonic currents in pcs in the presence of 5 μ M GABA. For **(E)** One-Way ANOVA followed by Bonferroni *post-hoc* test, ^***^p < 0.001; for **(F)** unpaired Student's *t*-test, ^*^p < 0.05. Exact values for *p* and *n* are given in the text.

We next applied the δ-GABA_A_R preferring agonist THIP. Since studies using THIP often report non-specific side-effects, we first evaluated in which concentration range THIP-induced effects could be considered δ-GABA_A_R-specific in the amygdala. According to our immunohistochemical stainings, BLA principal cells do not express δ-containing GABA_A_Rs and thus provide a suitable control. To rule out any presynaptic effects we recorded mIPSCs from BLA principal cells and analyzed mIPSC frequency, amplitude, and kinetic parameters in absence and presence of 1 μM THIP. No significant changes in any of these parameter could be detected (Table 1). Furthermore, application of 1 μM THIP had no effect on tonic currents and sIPSC parameters in BLA principal cells either (Table 1), yet 10 μM THIP clearly increased tonic currents in this cell type, indicative of a non-specific effect (data not shown). In pcs, application of 0.5, 1 and 10 μM THIP followed by bicuculline (25 μM) clearly led to a concentration-dependent effect on tonic currents with a significant effect at 1 and 10 μM compared to application of bicuculline alone (control: 0.148 ± 0.011 pA/pF, *n* = 25, vs. 0.5 μM THIP: 0.181 ± 0.044, *n* = 5, vs. 1 μM THIP: 0.322 ± 0.022 pA/pF, *n* = 9, vs. 10 μM THIP: 0.520 ± 0.081 pA/pF, *n* = 4, *p* > 0.05 for 0.5 μM THIP, *p* < 0.001 for both 1 and 10 μM THIP, One-Way ANOVA followed by Bonferroni *post-hoc* test) (Figures 4A,B,E). Baseline noise (12 ± 1 pA) was markedly increased in the presence of 1 μM THIP (49 ± 13%) and even more so in the presence of 10 μM THIP (145 ± 22%) (Figures 4A,B). Bicuculline reversed baseline noise to control values only in the presence of lower drug concentrations 0.5 and 1 μM THIP, but not in the presence of 10 μM, arguing further for non-specific effects evoked by the high drug concentration (Figures 4A,B). Analysis of sIPSC parameter of pcs in the presence and absence of 1 μM THIP revealed significantly smaller values for τ_2_, when sIPSCs were fitted with a double exponential function (Figure 4A_1_ and Table 1, *n* = 12, paired Student's *t*-test, *p* = 0.0177), an effect also observed by others in δ-GABA_A_R carrying cells (Drasbek et al., 2007). Due to the high baseline noise sIPSCs could not be analyzed after wash-in of 10 μM THIP.

To confirm the role of δ-containing GABA_A_Rs in carrying tonic currents in pcs, we repeated the experiments described above with the neurosteroid THDOC. THDOC is an endogenous allosteric modulator that has been reported to preferentially enhance δ-containing GABA_A_Rs at low concentrations in a cell-specific manner (Herd et al., 2007). Bath-application of 300 nM THDOC followed by 25 μM bicuculline led to a shift, albeit small, in I_hold_ in 4 out of 9 pcs. Yet after averaging the bicuculline effect, no significant difference was observed comparing tonic conductance in presence and absence of THDOC (300 nM THDOC 0.156 ± 0.015pA/pF, *n* = 9, vs. control 0.148 ± 0.011 pA/pF, *n* = 25, *p* > 0.05, One-Way ANOVA followed by Bonferroni *post-hoc* test) (Figures 4C,D). However, sIPSC kinetics were clearly affected by 300 nM THDOC, as a longer decay time was observed (Figure 4C_1_, Table 1, *n* = 9, τ 2: *p* = 0.040, τ total: *p* = 0.010, paired Student's *t*-test). This finding is in line with earlier studies, reporting a neurosteroid-induced prolongation of IPSC decay (Cooper et al., 1999; Lambert et al., 2003; Cope et al., 2005). As the efficacy of THDOC to modulate tonic GABAergic currents might vary strongly with the levels of ambient GABA present (Houston et al., 2012), we wondered if the observed inconsistency might be due to low ambient GABA levels. Indeed, adding 5 μM GABA to the bath solution prior to the application of 300 nM THDOC led to a consistent modulation in tonic currents by the neurosteroid compared to the effect induced by 5 μM GABA alone (5 μM GABA: 0.245 ± 0.017 pA/pF, *n* = 6, vs. 5 μM GABA + 300 nM THDOC: 0.344 ± 0.024 pA/pF, *n* = 6, *p* = 0.0151, unpaired Student's *t*-test, Figures 4D,E,F and Table 1) In line with the effect on tonic current we observed an inconsistent effect on baseline noise with THDOC alone, but a consistent increase in baseline noise with THDOC in the presence of 5 μM GABA (83 ± 26%) with GABA by itself already increasing baseline noise by 22 ± 12%.

**Figure 5:**
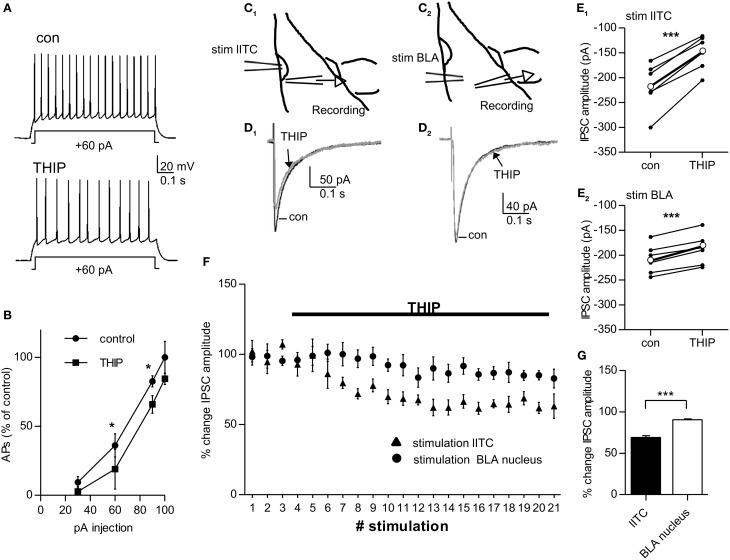
**1 μ M THIP decreases inhibitory output of lITC significantly and more strongly compared to that from BLA-residing interneurons. (A)** Pc injected with a +60 pA pulse for 0.8 s before (control) and after bath-application of 1 μ M THIP. The number of action potentials fired in response to the current pulse is calculated as % change in relation the number of action potentials fired with a +100pA injection under control conditions. **(B)** Summary graph of current injections into pcs before (control) and after application of 1 μ M THIP. Note the curve shift to the right under THIP with significant differences in action potential firing as response to +60 pA and +90 pA current injection pulse. **(C**_1_**–E**_1_**)** Extracellular stimulation with a ACSF-filled glass pipette in the lITC and recording of stimulated IPSCs in a BLA principal cells close the by the cluster before and after bath-application of 1 μ M THIP in presence of 2.5 mM kynurenic acid. C_1_ shows a schematic drawing of the experimental set-up **(D**_1_**)** Average traces of stimulated IPSC before and after application of THIP with stimulation of the lITC **(E**_1_**)** Summary plot showing the THIP effect on stimulated IPSC amplitude for single BLA principle cells (thin black dots and lines) and on average (thick black dot and line) with stimulation of the lITC. **(C**_2_**–E**_2_**)** The same as in **(C**_1_**–E**_1_**)** but in this case the stimulation electrode was placed in the BLA nucleus and stimulated IPSCs were recorded in a principal cell of the same nucleus. **(D**_2_**)** Average traces of stimulated IPSC before and after application of THIP with stimulation in the BLA nucleus **(E**_2_**)** Summary plot showing the THIP effect on stimulated IPSC amplitude for single BLA principle cells (thin black dots and lines) and on average (thick black dot and line) with stimulation of the BLA. **(F)** Comparison of the THIP effect on IPSC amplitude recorded in BLA principal cells with the stimulation electrode either placed in lITC or in BLA nucleus. **(G)** Summary graph comparing the change in stimulated IPSC amplitude for the two stimulation sites. IPSC amplitude is significantly more reduced when the IPSC is generated by lITC compared to BLA interneurons. For **(B)** paired Student's *t*-test, ^*^p < 0.05; for **(E**_1_**,E**_2_**)** paired Student's *t*-test ^***^p < 0.001, for **(G)** unpaired Student's *t*-test, ^*^p < 0.05. Exact values for *p* and *n* are given in the text.

If δ-containing GABA_A_Rs carry tonic currents in pcs, increasing δ-GABA_A_R activity should reduce action potential firing in these cells. To test this, action potentials were again evoked by stimulating pcs with depolarizing current steps (control: +30, +60, +90, +100 pA), and subsequently 1 μM THIP was washed in and current injections of the same magnitude was repeated. Pcs possess a strikingly low RMP, ranging in mouse on average between −78 mV (Marowsky et al., 2005) and −87 mV (Geracitano et al., 2007). Under our experimental conditions (32°C, 134.5 mM [Cl^−^] outside, 5 mM [Cl^−^] inside) the RMP of pcs is close to the reversal potential E_Cl−_ of −85 mV determined by the Nernst equation. Shifts in RMP were thus not expected to occur. 1 μM THIP still significantly reduced action potential firing in pcs (shown in % change in action potentials compared to control) and shifted the respective curve to the right, while the steepness of the curve was maintained (Injection of +30 and +100 pA: *p* > 0.05 for control vs. THIP effect; +60 pA injection: con 36.3 ± 5.6% vs. THIP 19.0 ± 0.8%, *p* = 0.046, *n* = 9; +90 pA injection: con 82.6 ± 2.6% vs. THIP 65.9 ± 2.1%, *n* = 9, *p* = 0.031, paired Student's *t*-test for all data points) (Figures 5A,B). Such a curve shift to the right is consistent with an increase in shunting inhibition (Mitchell and Silver, 2003), which would be expected to occur in the presence of a δ-preferring GABA_A_R agonist.

If a THIP-induced increase in tonic conductance leads to reduction in excitability of pcs, inhibition in target cells from pcs should be consequently decreased. In coronal slices the projection from pcs from the lITC onto BLA neurons are well preserved (Marowsky et al., 2005). We therefore placed a stimulation electrode on the dorsal part of the lITC and recorded stimulated IPSCs in BLA principal cells located ventrally in close vicinity to the cluster (Figures 5C_1_,D_1_). Bath-application of 1 μM THIP led to a clear reduction in the amplitude of the stimulated IPSC to 69.03 ± 2.02% (control: 216.2 ± 19.6 pA vs. THIP: 149.3 ± 14.4 pA, *n* = 6, *p* = 0.00026, paired Student's *t*-test) (Figure 5E_1_). As 1 μM THIP had no effect on sIPSC amplitude in BLA principal cells by itself (see Table 1), the observed decrease in IPSC amplitude can only be attributed to THIP-modulation of pcs residing in the lITC. For control, we placed the stimulation electrode in the BLA nucleus, where large δ-GABA_A_Rs-positive interneurons are abundant (see Figures 2A,E), and recorded from surrounding BLA principal cells (Figure 5C_2_). Under these conditions 1 uM THIP also showed a significant effect on the amplitude of stimulated IPSC amplitude, as it was decreased to 90.30 ± 1.79% (control: 207.8 ± 12.2 pA vs. THIP: 188.2 ± 12.9 pA, paired Student's *t*-test, *p* = 0.00031, *n* = 6) (Figures 5D_2_,E_2_). Comparison of the THIP effect after lITC and BLA stimulation revealed a highly significant difference between stimulation sites (lITC stimulation 69.03 ± 2.02% vs. BLA stimulation 90.30 ± 1.79%, *p* = 0.000144, unpaired Student's *t*-test) (Figures 5F,G). Taken together, the extracellular stimulation experiments confirmed that THIP is capable of strongly modulating output from pcs, most likely by increasing δ-GABA_A_R mediated tonic currents and shunting inhibition in these cells.

## Discussion

Here we provide converging immunohistochemical and electrophysiological evidence that δ-containing GABA_A_Rs mediate a substantial part of tonic currents in pcs of the amygdala. The δ-preferring agonist THIP increased tonic currents in pcs in a concentration-dependent manner; furthermore, THIP reduced action potential firing and the inhibitory output from lITC neurons onto BLA principal cells by 30%. Our immunohistochemical stainings revealed that δ-containing GABA_A_R expression in the LA and BLA nuclei is cell-type specific, confined to ITCs and a small subset of large GABAergic interneurons mostly residing in the BLA nucleus. The δ-subunit most often co-assembles with the α4-subunit to form GABA_A_Rs mediating tonic currents (Fritschy and Brunig, 2003; Farrant and Nusser, 2005). We found the α4-GABA_A_R subunit to be ubiquously expressed throughout LA and BLA nuclei including the ITCs, albeit overall to a lower degree compared to notoriously α4-subunit rich brain regions such as the thalamus (see e.g., Figure 3 in Marowsky et al., 2012). It is therefore plausible that α4/δ-containing GABA_A_Rs carry tonic currents in pcs. Incorporating a δ- instead of a γ2-subunit into a GABA_A_R decisively influences location, functional and pharmacological properties of the respective receptor. Indeed, δ-containing GABA_A_Rs were shown to be targeted almost exclusively to extrasynaptic sites, to have high affinity for GABA and a slow desensitization rate—all properties that render this receptor variant highly suitable for the mediation of a tonic conductance (Nusser et al., 1998; Nusser and Mody, 2002; Wei et al., 2003; Mtchedlishvili and Kapur, 2006).

The δ-preferring agonist THIP, also known as gaboxadol, is an artificial sleep-promoting and hypnotic drug (Krogsgaard-Larsen et al., 2004; Wafford and Ebert, 2006), which also showed antinociceptive properties in animal models of acute pain (Bonin et al., 2011). The drug was reported to have an EC_50_ of 6.3 μ M in α4δ β 3 receptors (Brown et al., 2002), and an EC_50_ of 40 μ M in α5β 3γ2 receptors, while EC_50_ values for other heterologous α1 and α3-containing GABA_A_Rs range from 143 to 499 μ M (Ebert et al., 1994). In our experiments, a THIP concentration as low as 0.5 μ M already enhanced tonic currents in 80% of pcs, while a higher concentration (1 μ M) led to a specific and substantial increase of tonic currents in all pcs recorded from and a clear increase in baseline noise. The fact that THIP showed a consistent effect in pcs at a concentration of 1 μ M strongly suggests the involvement of δ-carrying GABA_A_Rs, as comparative studies in δ KO- and WT-animals have shown that exclusively δ-subunits display such high sensitivity for THIP (Meera et al., 2011). Moreover, a concentration in the low micromolar range is considered clinically relevant, as it corresponds to plasma concentrations in humans after oral intake of 15 mg (Lund et al., 2006) and to brain levels in rats after an intraperitoneal dose of 6 mg/kg (Cremers and Ebert, 2007).

In contrast to THIP, the neurosteroid THDOC has been shown to act on synaptic as well as extrasynaptic receptors, yet the latter appear to be more sensitive to neurosteroid action than their synaptic counterparts (Herd et al., 2007). Overall, sensitivity to THDOC seems to vary considerably with cell type: while 10 nM of THDOC have been reported to enhance tonic currents in dentate gyrus granule cells (Stell et al., 2003), a minimum concentration of 200 nM THDOC was needed to observe potentiation of tonic currents in laminar II neurons in lumbar spinal cord slices (Bonin et al., 2011). In our preparation, a raise in ambient GABA level was required to observe a consistent effect on tonic currents with 300 nM THDOC.

Indeed, the strength of the THDOC effect seems strongly dependent on ambient GABA levels and these probably differ markedly between brain regions. Generally, neurosteroids modulation of extrasynaptic GABA_A_Rs is thought to rise in efficacy with increasing ambient GABA levels and is substantial with ambient GABA levels >100 nM (Houston et al., 2012). By contrast, the ability of THIP to potentiate tonic currents diminishes with increasing GABA levels. This is due to the fact that THIP acts a full agonist (or “superagonist”) at δ-containing GABA_A_Rs, while GABA is only a partial agonist at this receptor variant (Bianchi and Macdonald, 2003). Importantly, THIP and GABA compete for the same binding site, but affinity of extrasynaptic GABA_A_Rs is much higher for GABA than for THIP. Consequently, THIP efficiently enhances tonic conductance when ambient GABA levels are low, but fails to do so in presence of high ambient GABA concentrations, with GABA concentrations >250 nM completely abolishing the ability of THIP to enhance tonic inhibition (Houston et al., 2012). Given the fact that THIP substantially enhances tonic currents in pcs, while an increase in ambient GABA was required for a consistent THDOC effect to occur strongly indicates the presence of low ambient GABA levels in the respective cell clusters. However, these levels seem still sufficient to modulate the excitability in the electrotonically dense pcs (see Figure 1).

In general, ambient levels of GABA in slices are believed to be much lower than those existing *in vivo* (Lerma et al., 1986). Moreover, we used GAD67-GFP mice for proper identification of pcs. These mice lack one allele for the conversion from glutamate to GABA and consequently a 30% lower ambient GABA concentration was observed in the telencephalon from GAD67-GFP mice compared to WT littermates at embryonal day 17.5 (Morishima et al., 2010). As for *in vivo* levels of GABA, microdialysis studies have reported ambient GABA concentrations from 14 to 100 nM in the BLA nucleus of rats (Venton et al., 2006; Rea et al., 2009). These levels can be enhanced up to 400 nM during specific behavioral paradigms such as fear conditioning (Venton et al., 2006). THIP and THDOC effect *in vivo* might thus differ drastically from what we have observed in brain slice preparations.

To complicate matters further, the interaction of another neurosteroid, THP, with δ-containing GABA_A_Rs was reported to depend markedly on the age of experimental animals. In fact, during puberty THP rather inhibited α4β δ- GABA_A_Rs in female mice, probably by facilitating receptor desensitization and consequently reduced rather than increased tonic currents in CA1 hippocampal cells (Shen et al., 2007). Moreover, expression of the α4β δ- GABA_A_R variant in CA1 hippocampal cells was markedly increased during puberty in female mice. Even though, we used exclusively male mice for the complete set of experiments (aged 3–5 weeks), it is possible that neurosteroid effects in pcs will substantially vary with age and gender of the animal.

## Physiological implications

In presence of THIP, feedforward inhibition by lITC neurons onto BLA principal cells is reduced as well as inhibition generated from a subset of δ-carrying BLA interneurons. As net result, THIP most likely leads to an enhancement of excitation in the BLA nucleus and consequently anxiety-like behavior should be increased (Sanders and Shekhar, 1995). Moreover, given the dense innervation of pcs by cortical fibers and the presumed role of these cells in mediating cortical control over the amygdala, it is conceivable that in presence of THIP cortical control might be attenuated, facilitating anxiogenic output of the CeA. However, THIP also targets neurons in the CeA. Here, a subset of neurons possesses a δ-GABA_A_R mediated tonic conductance that is increased under THIP (Herman et al., 2013), which might counteract the anxiogenic action of THIP on pcs and rather leads to anxiolytic effects. Experimental evidence from animals and humans suggests, that THIP shows, if at all, weak anxiolytic effects (Hoehn-Saric, 1983; Saarelainen et al., 2008). Finally, δ-GABA_A_R targeting compounds including THIP and neurosteroids might possibly interfere with pcs-related processes such as fear extinction. Activation of δ-GABA_A_Rs on pcs decreases cell input resistance and the amplitude of subsequent excitatory post-synaptic amplitude is consequently reduced (shunting inhibition) with the result that depolarization might now be insufficient to enable the induction of extinction-related plasticity.

### Conflict of interest statement

The authors declare that the research was conducted in the absence of any commercial or financial relationships that could be construed as a potential conflict of interest.
